# Quenching of dynamic nuclear polarization by spin–orbit coupling in GaAs quantum dots

**DOI:** 10.1038/ncomms8682

**Published:** 2015-07-17

**Authors:** John M. Nichol, Shannon P. Harvey, Michael D. Shulman, Arijeet Pal, Vladimir Umansky, Emmanuel I. Rashba, Bertrand I. Halperin, Amir Yacoby

**Affiliations:** 1 Department of Physics, Harvard University, Cambridge, Massachusetts 02138, USA.; 2 Braun Center for Submicron Research, Department of Condensed Matter Physics, Weizmann Institute of Science, Rehovot 76100, Israel.

## Abstract

The central-spin problem is a widely studied model of quantum decoherence. Dynamic nuclear polarization occurs in central-spin systems when electronic angular momentum is transferred to nuclear spins and is exploited in quantum information processing for coherent spin manipulation. However, the mechanisms limiting this process remain only partially understood. Here we show that spin–orbit coupling can quench dynamic nuclear polarization in a GaAs quantum dot, because spin conservation is violated in the electron–nuclear system, despite weak spin–orbit coupling in GaAs. Using Landau–Zener sweeps to measure static and dynamic properties of the electron spin–flip probability, we observe that the size of the spin–orbit and hyperfine interactions depends on the magnitude and direction of applied magnetic field. We find that dynamic nuclear polarization is quenched when the spin–orbit contribution exceeds the hyperfine, in agreement with a theoretical model. Our results shed light on the surprisingly strong effect of spin–orbit coupling in central-spin systems.

## Abstract (web summary)

Dynamic nuclear polarization is the transfer of electronic angular momentum to nuclear spins and is a potential route for coherently manipulating spin in quantum information. Here, the authors show that spin–orbit coupling can quench dynamic nuclear polarization in a gallium arsenide quantum dot.

## Graphical abstract

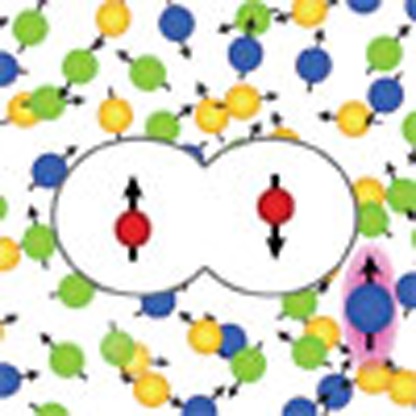

Dynamic nuclear polarization (DNP)^1^ occurs in many condensed matter systems, and is used for sensitivity enhancement in nuclear magnetic resonance^2^ and for detecting and initializing solid-state nuclear spin qubits^3^. DNP also occurs in two-dimensional electron systems^4^ via the contact hyperfine interaction. In both self-assembled^5^,^6^,^7^,^8^,^9^ and gate-defined quantum dots^10^,^11^,^12^,^13^, for example, DNP is exploited to prolong coherence times for quantum information processing. Closed-loop feedback^12^ based on DNP, in particular, is a key component in one- and two-qubit operations in singlet-triplet qubits^11^,^14^,^15^.

Despite the importance of DNP, it remains unclear what factors limit DNP efficiency in semiconductor spin qubits^16^. In particular, the relationship between the spin–orbit and hyperfine interactions^17^,^18^,^19^,^20^ has been overlooked in previous experimental studies of DNP in quantum dots, although several works have shown that the spin–orbit and hyperfine interactions contribute to spin relaxation^21^,^22^,^23^ under different conditions. It has been theoretically predicted, although not observed experimentally, that the spin–orbit interaction should limit DNP by providing a route for electron spin flips without corresponding nuclear spin flops^18^,^20^,^24^.

In this work, we show that spin–orbit coupling competes with the hyperfine interaction and ultimately quenches DNP in a GaAs double quantum dot^14^,^25^, even though the spin–orbit length is much larger than the interdot spacing. We use Landau–Zener (LZ) sweeps to characterize the static and dynamic properties of Δ_ST_(*t*), the coupling between the singlet *S* and *m*_*s*_=1 triplet *T*_+_, and the observed suppression of DNP agrees quantitatively with a theoretical model. In addition to improving basic understanding of DNP in semiconductors, these results will enable enhanced coherence times in semiconductor spin qubits by elucidating the experimental conditions under which DNP is most efficient^26^.

## Results

### Hyperfine and spin–orbit contributions to the *S*−*T*_+_ splitting

**Figure 1:**
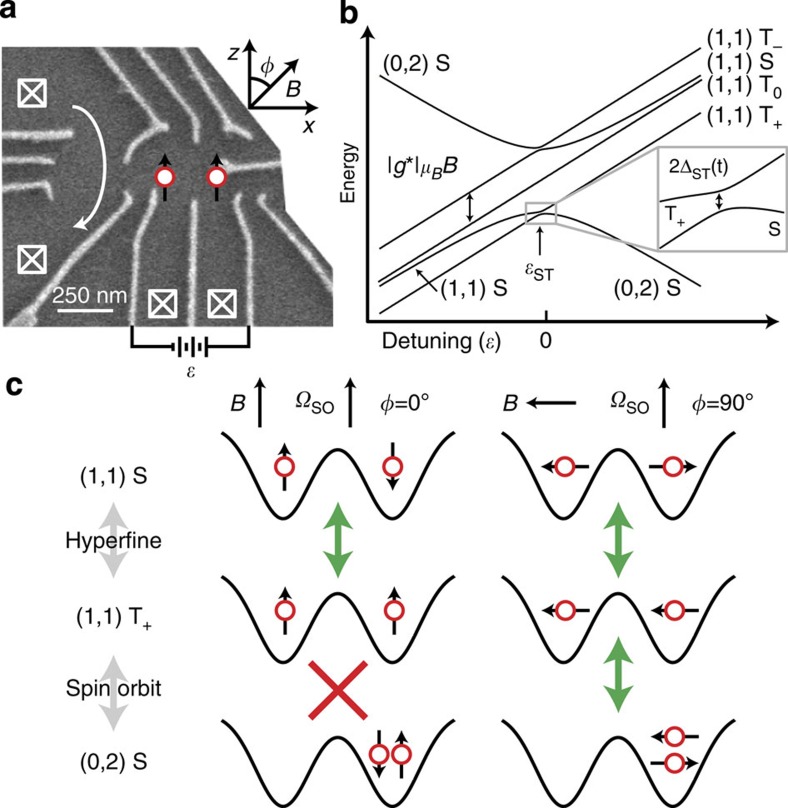
Experimental set-up. (**a**) Scanning electron micrograph of the double quantum dot. A voltage difference between the gates adjusts the detuning *ɛ* between the potential wells, and a nearby quantum dot on the left senses the charge state of the double dot. The gate on the right couples the double dot to an adjacent double dot, which is unused in this work. The angle between **B** and the *z* axis is *ϕ*. (**b**) Energy level diagram showing the two-electron spin states and zoom-in of the *S*−*T*_+_ avoided crossing. (**c**) The hyperfine interaction couples |(1,1)*S*〉 and |(1,1)*T*_+_〉 when the two dots are symmetric, regardless of the orientation of **B**, and the spin–orbit interaction couples |(0,2)*S*〉 and |(1,1)*T*_+_〉 when **B** has a component perpendicular to 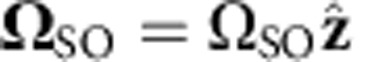, the effective spin–orbit field experienced by the electrons during tunnelling.

**Figure 2:**
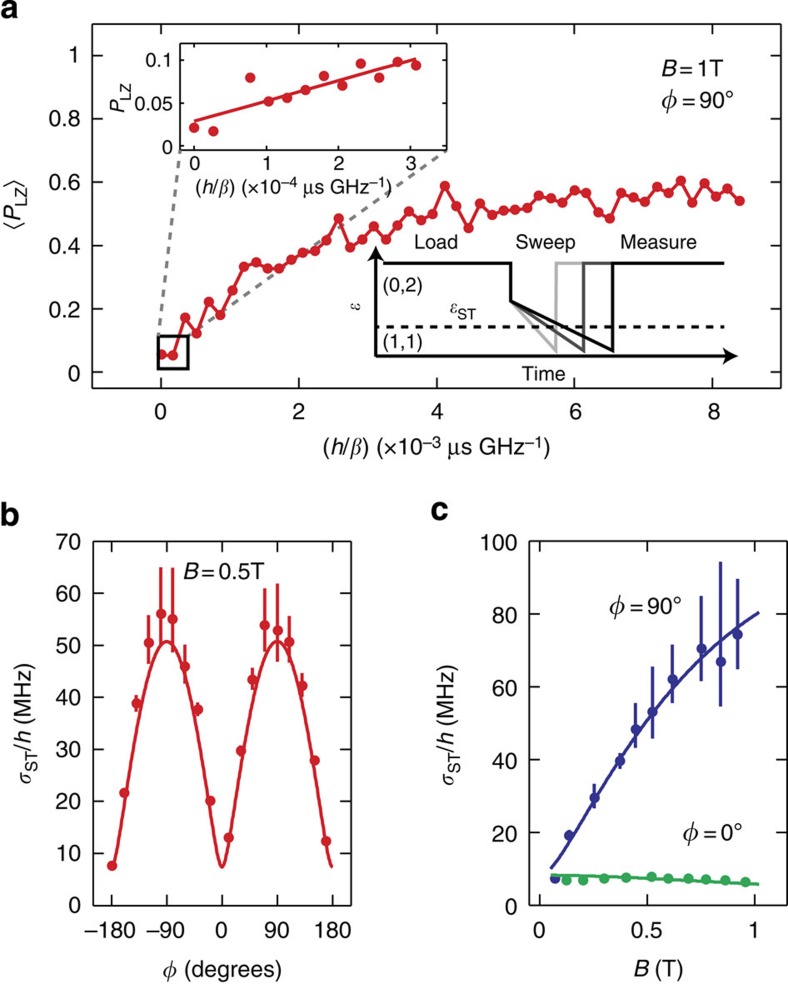
Measurements of *σ*_ST_. (**a**) Data for a series of LZ sweeps with varying rates, showing reduction in maximum probability due to charge noise. The horizontal axis is proportional to the sweep time. Upper inset: data and linear fit for fast sweeps such that 0<〈*P*_LZ_〉<0.1. Lower inset: in a LZ sweep, a |(0,2)*S*〉 state is prepared, and *ɛ* is swept through *ɛ*_*ST*_ (dashed line) with varying rates. Here *h*=2*πħ* is Planck's constant. (**b**) *σ*_ST_ versus *ϕ* (dots) and simulation (solid line). (**c**) *σ*_ST_ versus *B* for *ϕ*=0° and *ϕ*=90° (dots) and fits to equation (3) (solid lines). When *ϕ*=0°, Δ_SO_ is fixed at zero, and the only fit parameter is *σ*_HF_. When *ϕ*=90°, *σ*_HF_ is fixed at the fitted value, and Δ_SO_ is the only fit parameter (see Methods section). Error bars are fit errors.

Figure 1a shows the double quantum dot used in this work^14^,^25^. The detuning, *ɛ*, between the dots determines the ground-state charge configuration, which is either (1,1) (one electron in each dot) or (0,2) (both electrons in the right dot) as shown in Fig. 1b. To measure the *S*−*T*_+_ coupling, Δ_ST_(*t*), the electrons are initialized in |(0,2)*S*〉, *ɛ* is swept through the *S*−*T*_+_ avoided crossing at *ɛ*=*ɛ*_ST_, and the resulting spin state is measured (Fig. 2a). When *ɛ* ≈*ɛ*_ST_, we may describe the double quantum dot by an effective two-state Hamiltonian


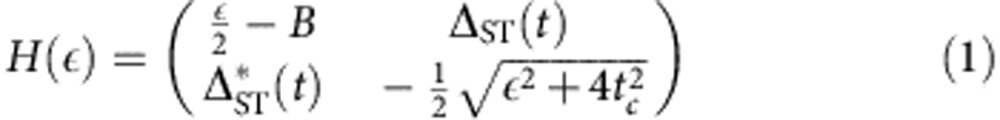


in the {|*T*_+_〉,|*S*〉} basis (Fig. 2b), where *t*_*c*_=23.1 μeV is the double-dot tunnel coupling and *B* is the external magnetic field strength. In the absence of any noise, the probability for an *S*−*T*_+_ transition is given by the LZ formula^27^,^28^:


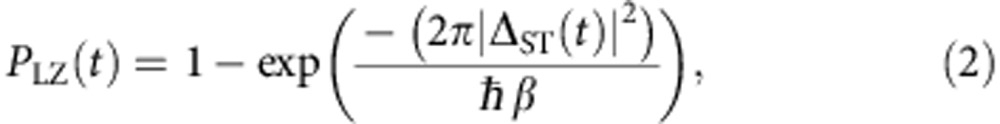


where *β*=*d*(*E*_*S*_−*E*_*T*+_)/d*t* is the sweep rate, with *E*_*S*_ and *E*_*T*+_ the energies of the *S* and *T*_+_ levels. Following the LZ sweep, we interpret the experimentally measured triplet return probability as the LZ probability, *P*_LZ_.

Equation (2) predicts transitions with near-unity probability for slow sweeps. For large magnetic fields, however, we experimentally observe maximum transition probabilities of ∼0.5. As discussed in Supplementary Note 1 and shown in Supplementary Figs 1 and 2, this reduction is a result of rapid fluctuations in the sweep rate arising from charge noise. Even in the presence of noise, however, the average LZ probability 〈*P*_LZ_(*t*)〉 can be approximated for fast sweeps as 
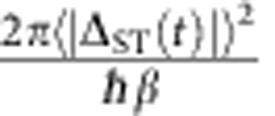
, which is identical to the leading order behaviour in *β*^−1^ of the usual LZ formula^29^. Here 〈⋯〉 indicates an average over the hyperfine distribution and charge fluctuations. To accurately measure 
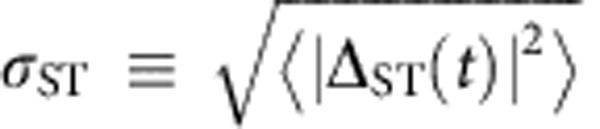
, we therefore fit 〈*P*_LZ_〉 versus *β*^−1^ to a straight line for values of *β* such that 0<〈*P*_LZ_〉<0.1 (Fig. 2a).

We first measure *σ*_ST_ versus *ϕ* at *B*=0.5 T (Fig. 2b), where *ϕ* is the angle between the magnetic field **B** and the *z* axis (Fig. 1a). *σ*_ST_ oscillates between its extreme values at 0° and 90° with a periodicity of 180°. Fixing *ϕ*=0° and varying *B*, we find that *σ*_ST_ decreases weakly with *B*, but when *ϕ*=90°, *σ*_ST_ increases steeply with *B*, reaching values >10 times that for *ϕ*=0°, as shown in Fig. 2c.

We interpret these results by assuming that both the hyperfine and spin–orbit interactions contribute to Δ_ST_(*t*) and by considering the charge configuration of the singlet state at *ɛ*_ST_ (Fig. 1b,c). The matrix element between *S* and *T*_+_ can be written as Δ_ST_(*t*)=Δ_HF_(*t*)+Δ_SO_. Δ_HF_(*t*)=g*μ_*B*_*δB*_⊥_(*t*) is the hyperfine contribution, which is a complex number, and it arises from the difference in perpendicular hyperfine field, 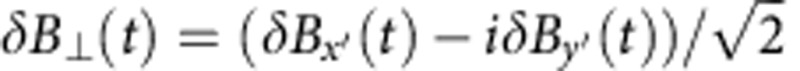, between the two dots^30^. Here 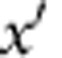 and 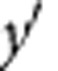 are coordinates perpendicular to **B**. (In the following, we set g*μ_*B*_=1 and give the hyperfine field strength in units of energy.) Δ_HF_(*t*) couples |(1,1)*S*〉 to |(1,1)*T*_+_〉 when the two dots are symmetric. Δ_SO_ is the spin–orbit contribution, which arises from an effective magnetic field 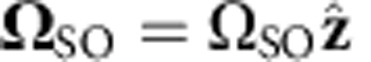 (see Methods section) experienced by the electron during tunnelling^17^. Only the component of Ω_SO_⊥**B** causes an electron spin flip. Δ_SO_ therefore couples |(0,2)*S*〉 to |(1,1)*T*_+_〉 when *ϕ* ≠0°, and Ω_SO_ is proportional to the double-dot tunnel coupling *t*_*c*_ (ref. 17), which is 23.1 μeV here. At *ɛ*_ST_, the singlet state |*S*〉 is a hybridized mixture: |*S*〉=cos*θ*|(1,1)*S*〉+sin*θ*|(0,2)*S*〉, where the singlet mixing angle 
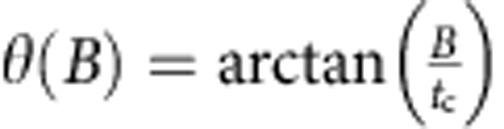
 approaches *π*/2 as *B* increases. Taking both *θ* and *ϕ* into account, we write^17^:


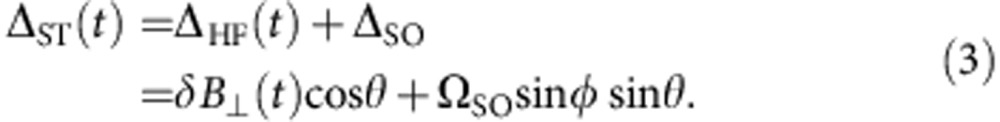


The data in Fig. 2b therefore reflect the dependence of Δ_ST_(*t*) on *ϕ* in equation (3). The data in Fig. 2c reflect the dependence of Δ_ST_(*t*) on *θ*. As *B* increases, *θ* also increases, and |*S*〉 becomes more |(0,2)*S*〉-like, causing Δ_HF_(*t*) to decrease. When *ϕ*=0°, Δ_SO_=0 for all *B*, but when *ϕ*=90°, Δ_SO_=Ω_SO_ sin*θ*, and *σ*_ST_ increases with *B*. Fitting the data in Fig. 2c allows a direct measurement of the spin–orbit and hyperfine couplings (see Methods section). We find 
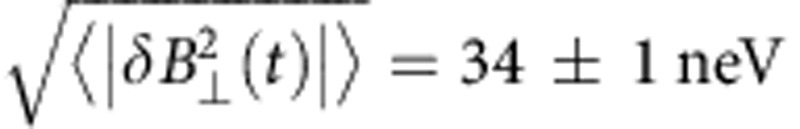
 and Ω_SO_=461±10 neV, corresponding to a spin–orbit length *λ*_SO_≈3.5 μm (refs 24, 31) (see Methods section), in good agreement with previous estimates in GaAs^32^,^33^,^34^.

### Spectral properties of the *S*−*T*_+_ splitting

We further verify that Δ_ST_(*t*) contains a significant spin–orbit contribution by measuring the dynamical properties of *P*_LZ_(*t*). A key difference between the spin–orbit and hyperfine components is that Δ_SO_ is static, whereas Δ_HF_(*t*) varies in time because it arises from the transverse Overhauser field, which can be considered a precessing nuclear polarization in the semiclassical limit^30^. To distinguish the components of Δ_ST_(*t*) through their time dependence, we develop a high-bandwidth technique to measure the power spectrum of *P*_LZ_(*t*).

**Figure 3:**
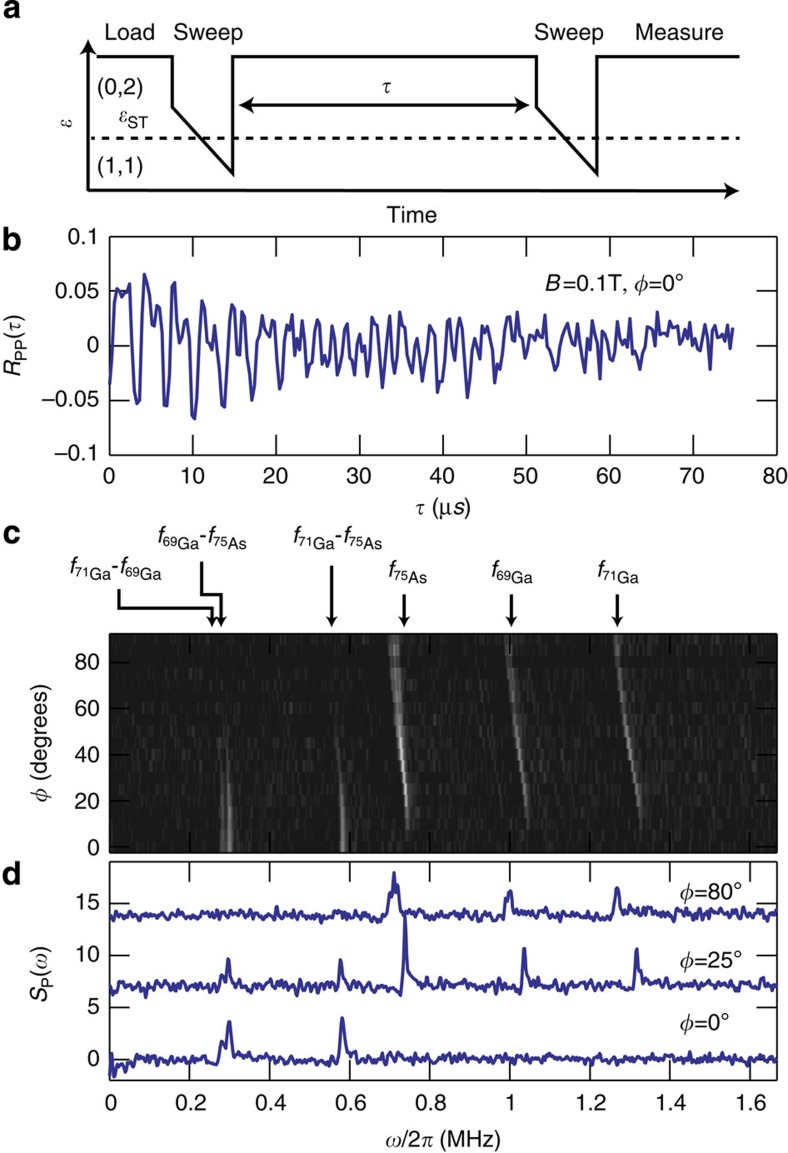
Correlations and power spectrum of *P*_LZ_(*t*). (**a**) Pulse sequence to measure *R*_PP_(*τ*) using two LZ sweeps. (**b**) *R*_PP_(*τ*) for *ϕ*=0° and *B*=0.1 T. The data extend to *τ*=200 μs, but for clarity are only shown to 75 μs here. (**c**) *S*_*P*_(*ω*) versus *ϕ* obtained by Fourier-transforming *R*_PP_(*τ*). At *ϕ*=0°, the differences between the nuclear Larmor frequencies are evident, but for |*ϕ*|>0°, the absolute Larmor frequencies appear, consistent with a spin–orbit contribution to *σ*_ST_. The reduction in frequency with *ϕ* is likely due to the placement of the device slightly off-centre in our magnet, and the reduction in amplitude of the difference frequencies occurs because the sweep rate *β* was increased with *ϕ* to maintain constant 〈*P*_LZ_〉 (see Methods section). (**d**) Line cuts of *S*_*P*_(*ω*) at *ϕ*=0°, 25° and 80°.

Instead of measuring the two-electron spin state after a single sweep, *ɛ* is swept twice through *ɛ*_ST_ with a pause of length *τ* between sweeps (Fig. 3a) (see Methods section). Assuming that Stückelberg oscillations rapidly dephase during *τ* (refs 15, 32), and after subtracting a background and neglecting electron spin relaxation, the time-averaged triplet return probability is proportional to *R*_PP_(*τ*)≡〈*P*_LZ_(*t*)*P*_LZ_(*t*+*τ*)〉, the autocorrelation of the LZ probability (Fig. 3b). Taking a Fourier transform therefore gives *S*_*P*_(*ω*), the power spectrum of *P*_LZ_(*t*) (Fig. 3c,d). For *P*_LZ_(*t*)<<1, *P*_LZ_(*t*)∝|Δ_ST_(*t*)|^2^, so 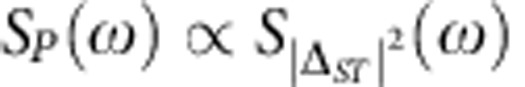, the power spectrum of |Δ_ST_(*t*)|^2^. This two-sweep technique allows us to measure the high-frequency components of *S*_*P*_(*ω*), because the maximum bandwidth is not limited by the quantum dot readout time.

Because it arises from the precessing transverse nuclear polarization, Δ_HF_(*t*) contains Fourier components at the Larmor frequencies of the ^69^Ga, ^71^Ga and ^75^As nuclei in the heterostructure, that is, 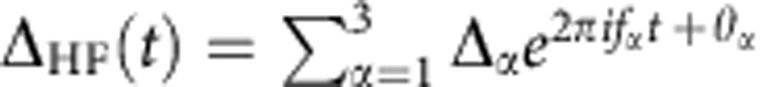, where *α* indicates the nuclear species, and the *θ*_*α*_ are the phases of the nuclear fields. Without spin–orbit interaction, 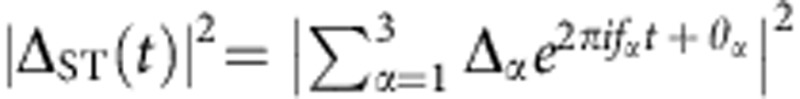 contains only Fourier components at the differences of the nuclear Larmor frequencies. With a spin–orbit contribution, however, |Δ_ST_(*t*)|^2^=|Δ_SO_+Δ_HF_(*t*)|^2^ contains cross-terms such as 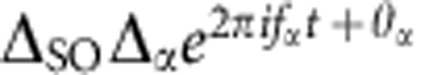 that give |Δ_ST_(*t*)|^2^ Fourier components at the absolute Larmor frequencies. A signature of the spin–orbit interaction would therefore be the presence of the absolute Larmor frequencies in *S*_*P*_(*ω*) for *ϕ* ≠0° (ref. 35).

Figure 3b shows *R*_PP_(*τ*) measured with *B*=0.1 T and *ϕ*=0°. Fig. 3c shows *S*_*P*_(*ω*) for 0°≤*ϕ*≤90°. At *ϕ*=0°, only the differences between the Larmor frequencies are evident, but as *ϕ* increases, the absolute nuclear Larmor frequencies appear, as expected for a static spin–orbit contribution to Δ_ST_(*t*). These results, including the peak heights, which reflect isotopic abundances and relative hyperfine couplings, agree well with simulations (Supplementary Fig. 3).

### Dynamic nuclear polarization

**Figure 4:**
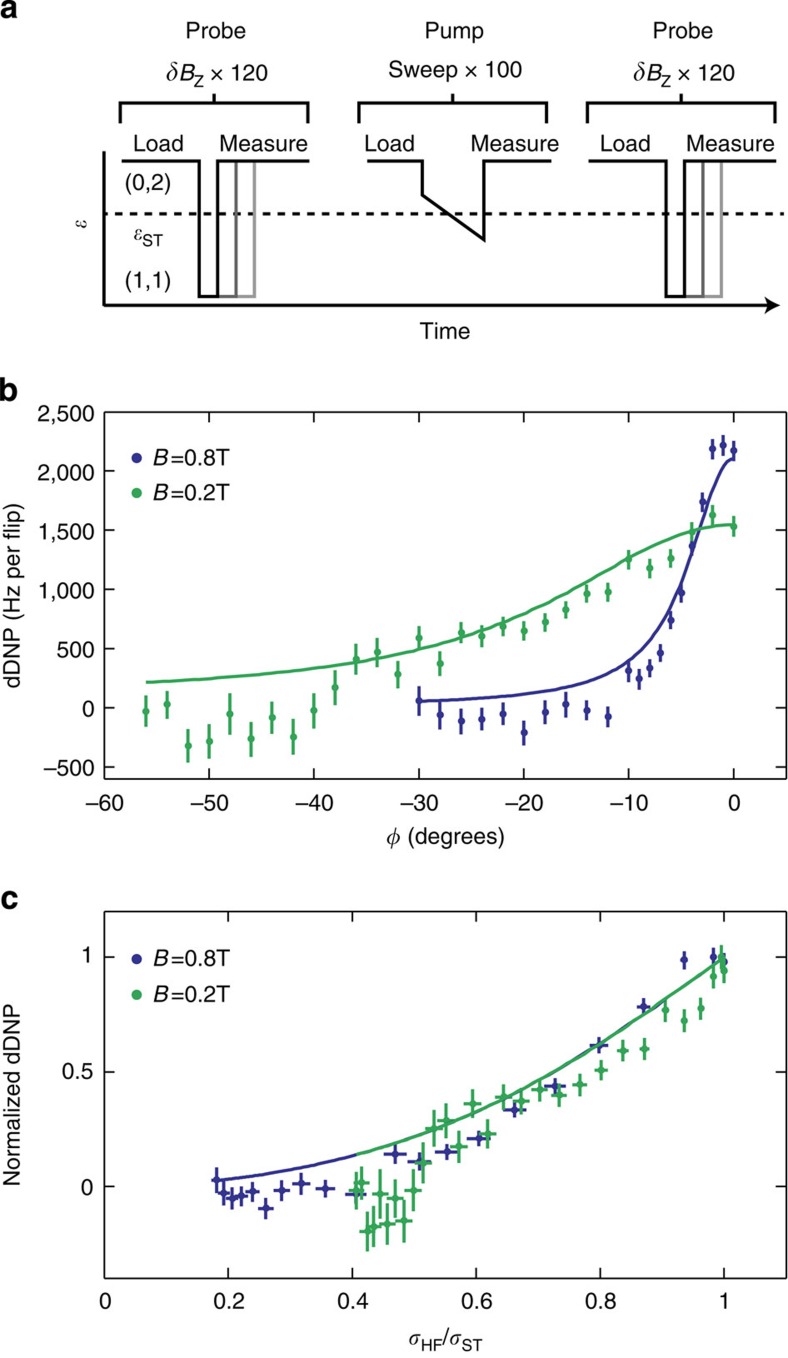
DNP quenching by spin–orbit coupling. (**a**) Protocol to measure DNP. *δB*_*z*_ is measured before and after 100 LZ sweeps by evolving the electrons around *δB*_*z*_. (**b**) dDNP versus *ϕ* at fixed 〈*P*_LZ_〉=0.4 for *B*=0.8 and 0.2 T, and theoretical curves (solid lines). dDNP is suppressed for |*ϕ*|>0 because of spin–orbit coupling. (**c**) Data and theoretical curves for fixed 〈*P*_LZ_〉 collapse when normalized and plotted versus *σ*_HF_/*σ*_ST_. Vertical error bars are statistical uncertainties and horizontal error bars are fit errors.

Having established the importance of spin–orbit coupling at the *S*−*T*_+_ crossing, we next investigate how the spin–orbit interaction affects DNP. Previous research has shown that repeated LZ sweeps through *ɛ*_ST_ increase both the average and differential nuclear longitudinal polarization in double quantum dots^11^. However, the reasons for left/right symmetry breaking, which is needed for differential DNP (dDNP), and the factors limiting DNP efficiency in general are only partially understood. Here we measure dDNP precisely by measuring *δB*_*z*_, the differential Overhauser field, using rapid Hamiltonian learning strategies^36^ before and after 100 LZ sweeps to pump the nuclei with rates chosen such that 〈*P*_LZ_〉=0.4 (see Methods section; Fig. 4a).

Figure 4b plots the change in *δB*_*z*_ per electron spin flip for *B*=0.2 and 0.8 T for varying *ϕ*. In each case, the dDNP decreases with |*ϕ*|. Because the spin–orbit interaction allows electron spin flips without corresponding nuclear spin flops, dDNP is suppressed as |Δ_SO_|=|Ω_SO_ sin*ϕ* sin*θ*| increases with |*ϕ*|. The reduction in dDNP occurs more rapidly at 0.8 T because *θ*, and hence Δ_SO_, are larger at 0.8 T than at 0.2 T. We gain further insight into this behaviour by plotting the data against *σ*_HF_/*σ*_ST_, where 
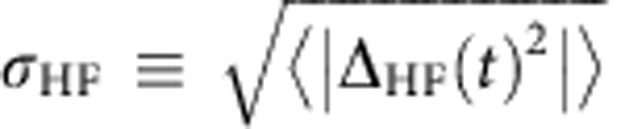
 (Fig. 4c). Plotted in this way, the two data sets show nearly identical behaviour, suggesting that the size of the hyperfine interaction relative to the total splitting primarily determines the DNP efficiency.

It is interesting to note that the peak DNP efficiency is less at *B*=0.2 T than at *B*=0.8 T. A possible explanation is that the electron–nuclear coupling becomes increasingly asymmetric with respect to the quantum dots at higher fields, because the singlet state becomes more |(0,2)*S*〉-like as *θ* increases^37^. The gradient build-up could also be due to an asymmetry in the size of the quantum dots^38^. As a result, we expect that the dDNP should be proportional to the total DNP, with a constant of proportionality that depends possibly on *B*, but not *β* or *ϕ*, in agreement with forthcoming theoretical and experimental work. We therefore explain our measurements of dDNP using a theoretical model in which we have computed the average angular momentum 〈*δm*〉 transfered to the ensemble of nuclear spins following a LZ sweep as:


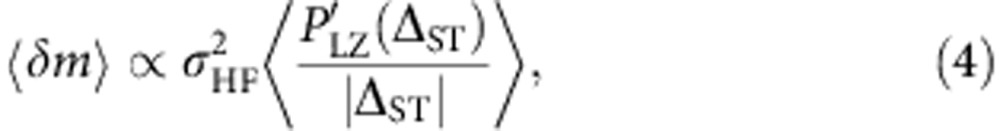


where 
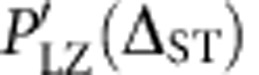
 is the derivative of the LZ probability with respect to the magnitude of the splitting. (See Supplementary Note 2 for more details.) Neglecting charge noise, we have the usual LZ formula (equation (2)), and equation (4) reduces to


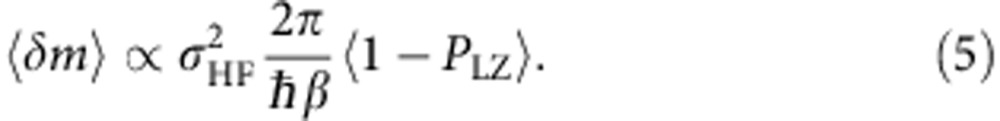


The data in Fig. 4b,c can therefore be understood in light of equation (5) because as the splitting *σ*_ST_ increases with |*ϕ*|, the sweep rate *β* was also increased to maintain a constant 〈*P*_LZ_〉. Because the hyperfine contribution *σ*_HF_ is independent of *ϕ*, 〈*δm*〉 therefore decreases. The data collapse in Fig. 4c can also be understood from equation (5), assuming constant Δ_ST_(*t*) and fixed *P*_LZ_. In this case, *β*∝|Δ_ST_|^2^, as follows from equation (2), and hence 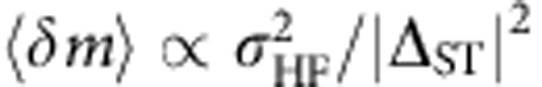. Measurements with fixed rate *β* also exhibit a similar suppression of dDNP (Supplementary Fig. 4 and Supplementary Note 2). In this case 〈*P*_LZ_〉 increases with |*ϕ*|, because of the increasing spin–orbit contribution to *σ*_ST_, and according to equation (5), 〈*δm*〉 therefore decreases.

The two theoretical curves in Fig. 4b,c are calculated using equation (5) multiplied by fitting constants *C*, which are different for the two fields, and agree well with the data. As discussed in Supplementary Note 2 and shown in Supplementary Fig. 5, we do not expect charge noise to modify the agreement between theory and data in Fig. 4b,c beyond the experimental accuracy. Finally, the peak dDNP value also approximately agrees with a simple calculation (Supplementary Note 3) based on measured properties of the double dot.

## Discussion

In summary, we have used LZ sweeps to measure the *S*−*T*_+_ splitting in a GaAs double quantum dot. We find that the spin–orbit coupling dominates the hyperfine interaction and quenches DNP for a wide range of magnetic field strengths, unless the magnetic field is oriented such that **B**||**Ω**_SO_. A misalignment of **B** to **Ω**_SO_ by only 5° at *B*=1 T can reduce the DNP rate by a factor of two, and DNP is completely suppressed for a misalignment of 15°. The techniques developed here are directly applicable to other quantum systems such as InAs or InSb nanowires and SiGe quantum wells, where the spin–orbit and hyperfine interactions compete. On a practical level, these results will improve coherence times in gate-defined quantum dot spin qubits by enabling more efficient DNP^12^, and the high-bandwidth correlation measurements demonstrated here offer a new tool to investigate nuclear dynamics in semiconductors. On a fundamental level, our findings suggest avenues of exploration for improved *S*−*T*_+_ qubit operation^32^ and underscore the importance of the spin–orbit interaction in the study of nuclear dark states^37^,^38^ and other mechanisms that limit DNP efficiency in central-spin systems.

## Methods

### Device details

The double dot is fabricated on a GaAs/AlGaAs hetereostructure with a two-dimensional electron gas located 90 nm below the surface. Au/Pd depletion gates are used to define the double-dot potential. The double dot is cooled in a dilution refrigerator to a base temperature of ∼50 mK. The double-dot axis is aligned within ≈5° of either the 
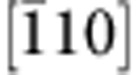
 or [110] axes of the crystal, but we do not know which. In the latter case, both the Rashba and Dresselhaus spin–orbit fields are aligned with the *z* axis, and their magnitudes add^17^. In the former case, the Rashba and Dresselhaus contributions are also aligned with the *z* axis, but their magnitudes subtract. Because the spin–orbit field is aligned with the *z* axis in each case, we do not expect the orientation of the double dot to qualitatively change our results. When *ϕ*=90°, *B* lies in the plane of the crystal and parallel to the double-dot axis.

### Measuring *σ*_ST_, Δ_SO_ and *σ*_HF_

In order to extract *σ*_ST_, we fit the measured 〈*P*_LZ_〉 versus *β* to a function of the form 
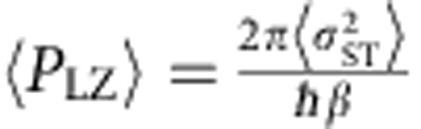
. We calibrate the sweep rate *β* using the spin-funnel technique^25^. We extract the spin–orbit and hyperfine strengths by fitting the data in Fig. 2c to a function of the form 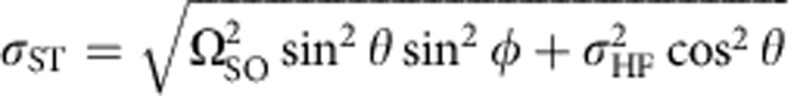, with Ω_SO_ and *σ*_HF_ as fit parameters. The singlet mixing angle 
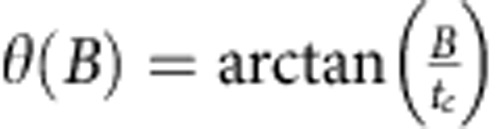
 is computed using the measured double-dot tunnel coupling *t*_*c*_=23.1 μeV.

Δ_SO_ is held at 0 when fitting data for *ϕ*=0° to determine the hyperfine coupling. We also exclude data points for *B*<0.2 T in the fit, as the hyperfine contribution appears to decrease at very low fields. We determine the spin–orbit length as 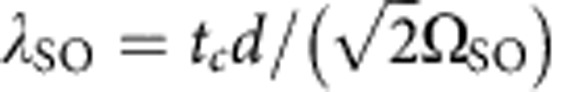 (refs 24, 31), where *t*_*c*_ is the interdot tunnel coupling, and *d*≈100 nm is half of the interdot spacing. The curve in Fig. 2b is a simulation, not a fit, and is generated using the same equation with the fitted values of Δ_SO_ and *σ*_HF_.

### Measuring *R*_PP_(*τ*)

Here we derive the triplet return probability after two consecutive LZ sweeps with a pause of length *τ* in between. In experiments, both sweeps were in the same direction, and *ɛ* was held in the (0,2) region between sweeps (Fig. 3a). If the first LZ sweep takes place at time *t* with probability *P*_LZ_(*t*), the probability for the two electrons to be in the *T*_+_ state is *P*_LZ_(*t*), whereas the probability to be in the *S* state is 1−*P*_LZ_(*t*). Then, the detuning is quickly swept into the (0,2) region, where electron spin dephasing occurs rapidly, and there is negligible *T*_+_ occupation in thermal equilibrium because the *S* and *T*_+_ states are widely separated in energy. After a wait of length *τ*, but before the second sweep, the triplet population is 
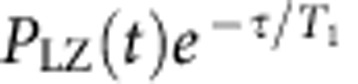
 and the singlet population is 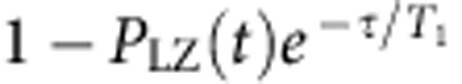, where *T*_1_ is the electron relaxation time. After the second sweep, the triplet occupation probability is


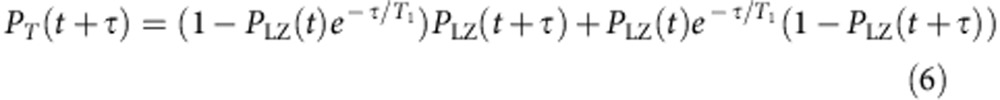



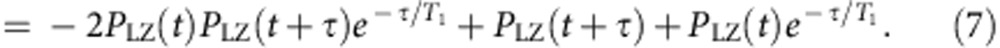


The second and third terms in equation (7) vary slowly with *τ*. Experimentally, these terms are found by fitting the measured triplet probability to an exponential with an offset and are subtracted. When *T*_1_>>*τ*, relaxation can be neglected, and the predicted time-averaged signal is 〈*P*_*T*_(*t*+*τ*)〉∝*R*_PP_(*τ*), where *R*_PP_(*τ*)≡〈*P*_LZ_(*t*)*P*_LZ_(*t*+*τ*)〉, the autocorrelation of the LZ probability. When *ϕ*=0°, *T*_1_>>*τ*_max_=200 μs, where *τ*_max_ is the largest value of *τ* measured. The shortest relaxation time *T*_1_≈100 μs in these experiments time occurs when *ϕ*=90°, which is consistent with spin–orbit-induced relaxation^23^.

The effect of *T*_1_ relaxation is to multiply the measured correlation by an exponentially decaying window, which reduces the spectral resolution of the Fourier transform, but does not shift the frequency of the observed peaks. We expect statistical fluctuations in the amplitude of the hyperfine field to affect the spectrum in a similar way, although we expect this effect to be less than that of electron relaxation. The raw data, (Fig. 3b) consisting of 667 points (each a result of two sweeps with a 40% chance of a LZ transition), spaced by 300 ns, were zero padded to a size of 1,691 points to smooth the spectrum, and a Gaussian window with time constant 150 μs was applied to reduce the effects of noise and ringing from zero padding before Fourier transforming.

The magnetic resonance frequencies in Fig. 3c decrease with *ϕ*. The inhomogeneity of the x-coil in our vector magnet is 1.6% at 0.6 cm offset from the centre. Thus, the field could easily be reduced by >3% for a misplacement of the sample by 1 cm from the magnet centre. We have simulated the data in Fig. 3c in the main text based on the measured hyperfine and spin–orbit couplings and the known sweep rates. Assuming a 4.4% reduction in the field from the x-coil, we obtain good agreement between theory and experiment (Supplementary Fig. 3). The difference frequencies also decrease in strength with increasing *ϕ*, which happens because the sweep rate *β* was increased with *ϕ* to maintain constant 〈*P*_LZ_〉. This effect can be understood for fast sweeps, where the amplitudes of the difference frequencies should scale as Δ_1_Δ_2_/*β*, where the subscripts indicate different nuclear species.

We argued in the main text that only the difference frequencies should appear in the spectrum *S*_*P*_(*ω*) without spin–orbit coupling by considering the time dependence of |Δ_ST_(*t*)|^2^ and because 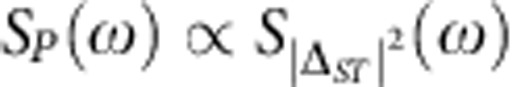 when *P*_LZ_(*t*)<<1. Since *P*_LZ_(*t*) contains only even powers of |Δ_ST_(*t*)|, *S*_*P*_(*ω*) can generally be expressed in terms of differences of the resonance frequencies, but will not contain the absolute frequencies in the absence of spin–orbit coupling, regardless of the value of *P*_LZ_(*t*).

### Measuring *δB*_*z*_

We measure *δB*_*z*_ by first initializing the double dot in the |(0,2)*S*〉 state and then separating the electrons by rapidly changing *ɛ* to a large negative value^25^. When the electrons are separated, the exchange energy is negligible, and the magnetic field gradient *δB*_*z*_ drives oscillations between |*S*〉 and |*T*_0_〉. In our experiments, we measure the two-electron spin state for 120 linearly increasing values of the separation time. The resulting single-shot measurement record is thresholded, zero padded and Fourier transformed. The frequency corresponding to the peak in the resulting Fourier transform is chosen as the value of *δB*_*z*_. This technique is related to a previously described rapid Hamiltonian estimation technique^36^.

## Additional information

**How to cite this article:** Nichol, J. M. *et al*. Quenching of dynamic nuclear polarization by spin–orbit coupling in GaAs quantum dots. *Nat. Commun.* 6:7682 doi: 10.1038/ncomms8682 (2015).

## Supplementary Material

Supplementary Information Supplementary Figures 1-5, Supplementary Notes 1-3 and Supplementary References
