# Supplementary material for: Quenching of dynamic nuclear polarization by spin–orbit coupling in GaAs quantum dots
Source: Nat Commun. 2015 Jul 17;6:7682. doi: 10.1038/ncomms8682 (PMC4518271; doi:10.1038/ncomms8682)
Supplement: Supplementary Information — Supplementary Figures 1-5, Supplementary Notes 1-3 and Supplementary References [file ncomms8682-s1.pdf]

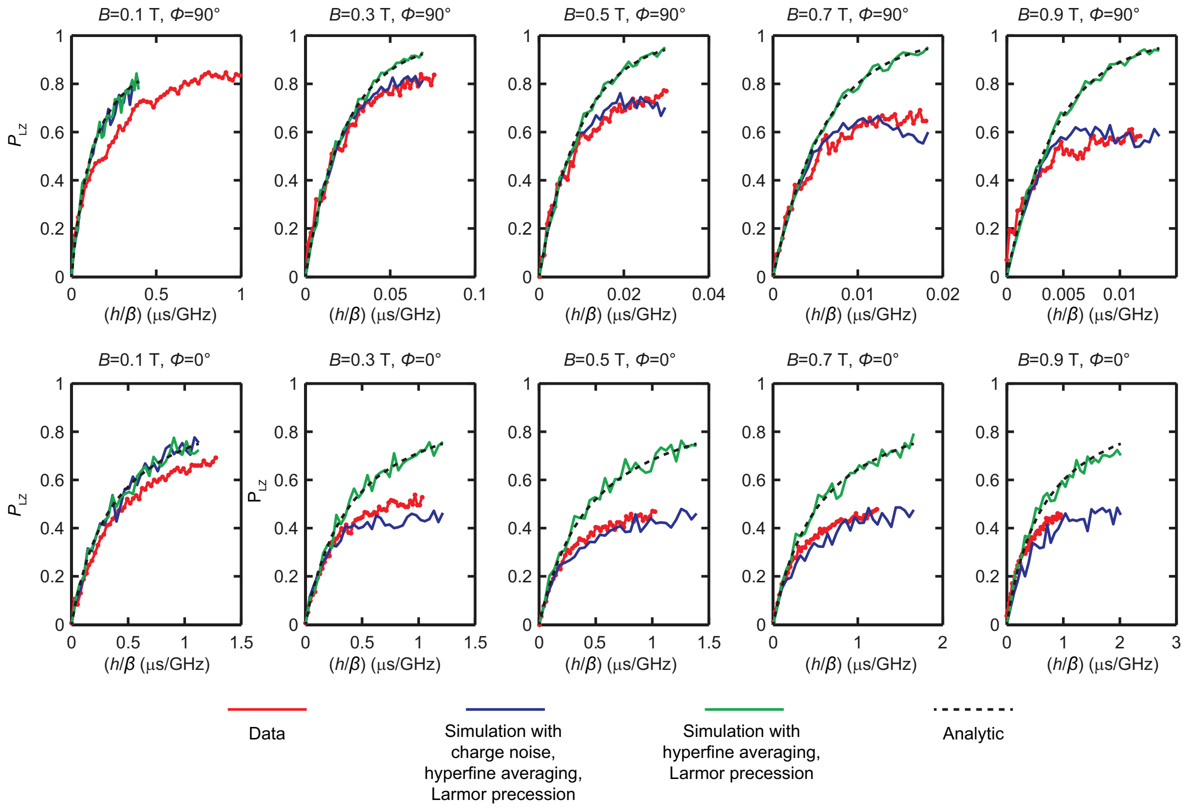

Supplementary Figure 1. Comparison of LZ data and simulations. Each panel shows data and simulations for a different magnetic field strength and orientation. Red curves are experimental data for a series of LZ sweeps with varying rates. Blue curves are simulated data including charge noise, hyperfine averaging, and nuclear Larmor precession for the calculated value of the splitting corresponding to the red curves. Green curves are simulated data with hyperfine averaging and Larmor precession for the same value of the splitting as the blue curves. Black curves are calculated via Supplementary Equation (4) using the same value of the splitting. In all panels, the y axis is $\langle P_{LZ} \rangle$, and the x axis is h/β (μs/GHz). Here $h = 2\pi\hbar$ is Planck's constant.

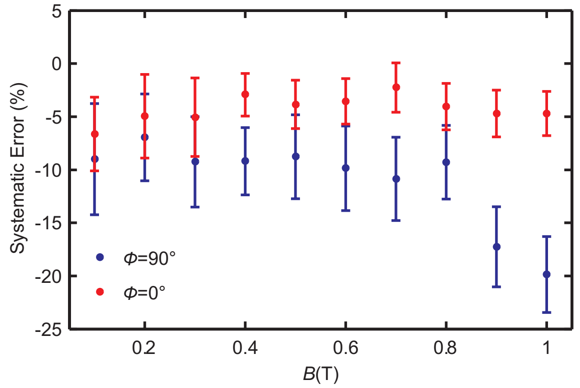

Supplementary Figure 2. Fitting error. We compute the fitting error by simulating $\langle P_{LZ} \rangle$ for the calculated splitting at each of the magnetic field configurations in the presence of charge noise. The simulated $\langle P_{LZ} \rangle$ vs β^{-1} is fitted to a straight line for $0 < \langle P_{LZ} \rangle < 0.1$, and the fitted value of the splitting is subtracted from the value chosen for the simulation. The difference is then divided by the simulated value of the splitting. Error bars are fit errors.

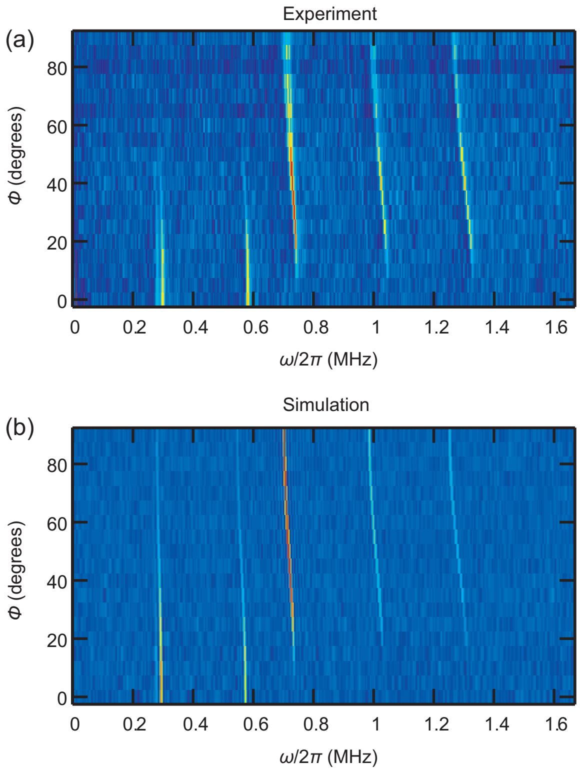

Supplementary Figure 3. Simulations of $S_P(\omega)$. (a) Experimental data. (b) Numerical simulation taking into account known sweep rates, nuclear magnetic resonance frequencies, hyperfine couplings, and a 4.4% reduction in field in the x direction. The expected frequencies at $B = 0.1$ T are $f_{^{69}Ga} = 1.0248$ MHz, $f_{^{71}Ga} = 1.302$ MHz, and $f_{^{75}As} = 0.7315$ MHz.

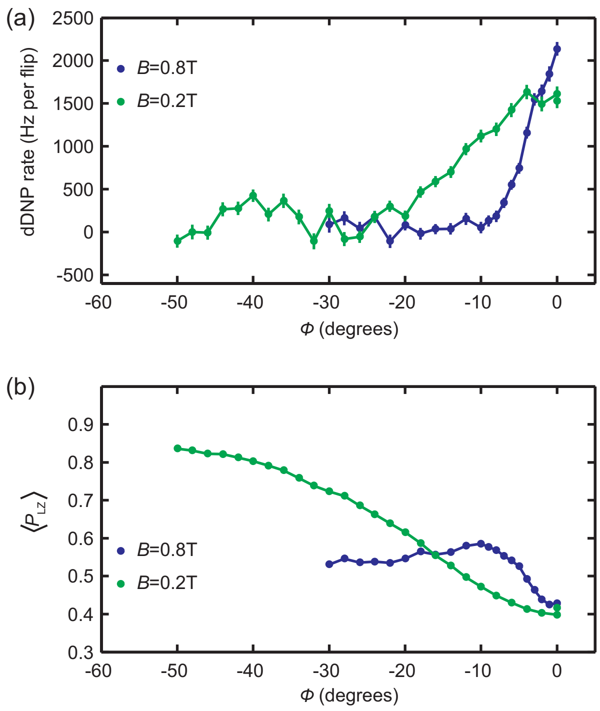

Supplementary Figure 4. DNP quenching with fixed sweep rate. (a) dDNP vs ϕ at $B = 0.2$ T and $B = 0.8$ T. For each field, the sweep rate β was chosen to give $\langle P_{LZ} \rangle = 0.4$ at $\phi = 0°$ and then was held constant for $\phi \neq 0°$. (b) As $|\phi|$ increases, σ_{ST} increases. As a result, $\langle P_{LZ} \rangle$ also increases and DNP is suppressed, according to Supplementary Equation (18). Error bars are statistical uncertainties. Lines between points serve as a guide to the eye.

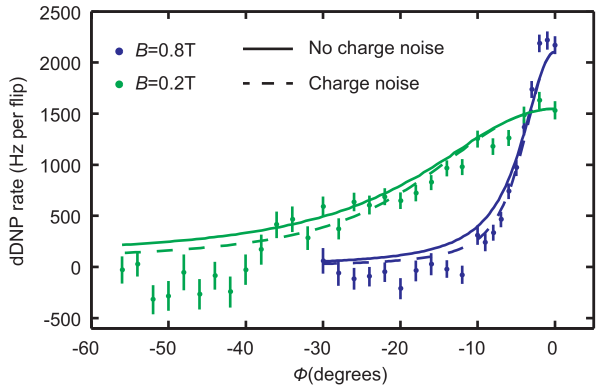

Supplementary Figure 5. The effect of charge noise on dDNP. The data and solid lines are the same as in Fig. 4 in the main text, and the dashed lines are the theoretical estimates for dDNP in the presence of charge noise. The dashed and solid lines are normalized to the same values at $\phi = 0°$. Error bars are statistical uncertainties.

SUPPLEMENTARY NOTE 1. MEASURING σ_{ST}

Here we describe the fitting procedure to extract σ_{ST}. The experimentally measured quantity is the average triplet occupation probability $\langle P_T \rangle$, which we interpret as the average Landau-Zener (LZ) probability $\langle P_{LZ} \rangle$, at the end of a sweep. Here $\langle \cdots \rangle$ indicates an average over the hyperfine distribution and charge fluctuations for the same nominal sweep parameters. We calibrate the rate $\beta = d(E_S - E_{T_+})/dt$ using the spin-funnel technique [1] and assume a linear change in the $S - T_+$ splitting near the avoided crossing.

$\Delta_{HF}(t)$ varies in time because of the nuclear Larmor precession and statistical fluctuations in the magnitude of the nuclear polarizations. We argue that both types of hyperfine fluctuations occur on time scales much longer than LZ transitions and can be treated as quasi-static. In typical experiments, the $S - T_+$ splitting is swept through approximately 5 GHz in less than 1 μs. For splittings of order 10 MHz, the total time spent near the avoided crossing is less than 10 ns, which is much faster than the nuclear Larmor period at 1 T, roughly 100 ns. Furthermore, during 1 μs, the nuclear polarization diffuses by approximately 7 kHz [2], which is 3 orders of magnitude smaller than σ_{HF}. We therefore assume that the splitting is constant during a single sweep. Numerical simulations discussed below also support the hypothesis that nuclear Larmor precession does not significantly affect $\langle P_T \rangle$ for the sweep rates used here (Supplementary Fig. 1).

In the absence of hyperfine or charge fluctuations, the probability for a transition is given by the LZ formula: $P_{LZ}(t) = 1 - \exp(-2\pi|\Delta_{ST}(t)|^2/(\hbar\beta))$ [3]. Neglecting high-frequency charge noise, the exact form of the LZ probability averaged over the hyperfine distribution can be computed. Let the total splitting be $\Delta_{ST} = \Delta_{HF} + \Delta_{SO}$. We take Δ_{SO} to be the constant, real spin-orbit part and Δ_{HF} the complex hyperfine contribution. Assuming that the real and imaginary parts of Δ_{HF} (u and v, respectively) are Gaussian-distributed around zero such that the root-mean-square hyperfine splitting is σ_{HF}, the probability distribution for the splitting to have magnitude $\Delta = |\Delta_{ST}|$ is

$$p(\Delta) = \frac{1}{\pi\sigma_{HF}^2}\int_{-\infty}^{\infty} du \int_{-\infty}^{\infty} dv\, e^{-\frac{u^2+v^2}{\sigma_{HF}^2}}\,\delta\left(\Delta - \sqrt{(\Delta_{SO}+u)^2+v^2}\right) \tag{1}$$

$$= \frac{2\Delta}{\sigma_{HF}^2} e^{-\frac{\Delta^2+\Delta_{SO}^2}{\sigma_{HF}^2}} I_0(2\Delta\Delta_{SO}/\sigma_{HF}^2), \tag{2}$$

where I_0 is the zeroth-order modified Bessel function of the first kind. Note that when $\Delta_{SO} =$

0, Supplementary Equation (2) reduces to the familiar distribution $p(\Delta) = \frac{2\Delta}{\sigma_{HF}^2} e^{-\Delta^2/\sigma_{HF}^2}$ [4]. Integrating the LZ probability over this distribution yields the average LZ probability $\langle P_{LZ} \rangle$:

$$\langle P_{LZ} \rangle = \int_0^\infty d\Delta \left(1 - \exp\left(-\frac{2\pi\Delta^2}{\hbar\beta}\right)\right) p(\Delta) \tag{3}$$

$$= 1 - Q \exp\left(-\frac{2\pi\Delta_{SO}^2}{\hbar\beta} Q\right), \tag{4}$$

with

$$Q = \frac{1}{1 + \frac{2\pi\sigma_{HF}^2}{\hbar\beta}}. \tag{5}$$

Note that this result agrees with another derivation [5]. Note also that to leading order in β^{-1}, $\langle P_{LZ} \rangle \approx 2\pi \left(\Delta_{SO}^2 + \sigma_{HF}^2\right) / \hbar\beta$.

The average triplet return probability $\langle P_T \rangle$ may be modified due to effects of charge noise on the defining gates or in the two-dimensional electron gas itself. High-frequency charge noise in double quantum dots has recently been identified as a major source of decoherence [6]. In the current setting, corrections to $\langle P_T \rangle$ should occur, because charge fluctuations lead to time-dependent variations in $S - T_+$ detuning $E_S - E_{T_+}$, on top of the linear time-dependence due to the prescribed sweep rate β. Additionally, charge fluctuations can add noise to the off-diagonal coupling $\Delta_{ST}(t) = \delta B_\perp(t) \cos\theta + \Omega_{SO} \sin\phi \sin\theta$, because the singlet mixing angle $\theta = \tan^{-1}\left(\frac{\epsilon + \sqrt{\epsilon^2 + 4t_c^2}}{2t_c}\right)$ depends on ϵ. (Here $t_c = 23.1$ μeV is the double-dot tunnel coupling.) As discussed below, however, the noise in Δ_{ST} should have much less effect than the detuning noise for the magnetic fields studied here.

We observe that for high magnetic fields and slow sweeps, the maximum LZ probability falls to 0.5 as shown in Supplementary Fig. 1. It was previously noted that strong detuning noise can have such an effect [7]. To confirm that charge noise causes the probability reduction, we have performed Monte Carlo simulations of the Schrödinger equation for symmetric double dots undergoing LZ sweeps, including the effects of wide-band charge noise, nuclear Larmor precession, and averaging over the hyperfine distribution. The results of the simulations and experimental data are shown in Supplementary Fig. 1. We generate random charge noise with power spectrum $14 \times 10^{-14} \, \frac{\mathrm{V}^2}{\mathrm{Hz}} \left(\frac{1\mathrm{Hz}}{f}\right)^{0.7}$ for $f < 1$ GHz, and 0 otherwise. We generate the Fourier transform of the charge noise time record by picking the amplitude corresponding to the chosen power spectrum and a random phase for each frequency f in the desired range. We then perform an inverse Fourier transform to obtain

the charge noise time record. The spectrum we chose corresponds to a noise amplitude of 3 nV/$\sqrt{\text{Hz}}$ at $f = 1$ MHz, which is approximately the measured level of charge noise in the double dot used here. Note that we have extrapolated the $f^{-0.7}$ frequency dependence that was previously measured to $f = 1$ MHz in ref. [6] up to $f = 1$ GHz in these simulations. However, one expects the results to be most sensitive to noise in the range of 10-100 MHz, corresponding to the size of the splitting. The ϵ-dependent Hamiltonian used in these simulations was

$$H(\epsilon) = \begin{pmatrix} \frac{\epsilon}{2} - B & \delta B_\perp(t)\cos\theta + \Omega_{SO}\sin\phi\sin\theta \\ \delta B_\perp^*(t)\cos\theta + \Omega_{SO}\sin\phi\sin\theta & -\frac{1}{2}\sqrt{\epsilon^2 + 4t_c^2} \end{pmatrix} \tag{6}$$

in the $\{|T_+\rangle, |S\rangle\}$ basis. Linear ϵ sweeps through the $S - T_+$ crossing $\epsilon_{ST} = \frac{B^2 - t_c^2}{B}$ were used in the simulation to replicate the actual experiments. For each strength and orientation of the magnetic field, θ was calculated at ϵ_{ST} using the measured tunnel coupling, and the fitted values of the spin-orbit and hyperfine couplings from the main text were used to compute the splitting. We assumed a lever arm of 10 to convert the voltage noise on the quantum dot gates to ϵ noise.

The simulated LZ curves with charge noise agree well with the data as shown in Supplementary Fig. 1. The same simulations including averaging over the hyperfine distribution and nuclear Larmor precession, but without charge noise, show very little reduction in probability compared with the analytic result, Supplementary Equation (4), supporting the hypothesis that charge noise is responsible for most of the observed probability reduction. A key feature in these experiments is the decreasing maximum probability with increasing magnetic field. We can understand that this trend occurs because the effect of charge noise on the Landau-Zener probability is controlled by the fluctuation in the energy splitting $\delta E(\epsilon)$ produced by a given fluctuation in the detuning ϵ, which is proportional to $\frac{dE(\epsilon)}{d\epsilon}|_{\epsilon=\epsilon_{ST}}$. Since $E(\epsilon) = \frac{\epsilon}{2} - B + \frac{1}{2}\sqrt{\epsilon^2 + 4t_c^2}$, the magnitude of $\frac{dE(\epsilon)}{d\epsilon}|_{\epsilon=\epsilon_{ST}}$ increases sharply with increasing magnetic field.

Even in the presence of noise, however, the average LZ probability in the limit of fast sweeps is still $2\pi|\Delta_{ST}(t)|^2/\hbar\beta$, which is identical to the leading order behavior of the usual LZ formula, as shown in section 3.1 of ref. [7]. Replacing the LZ formula in Supplementary Equation (4) by its leading order behavior, and performing the integration over the quasi-

static distribution gives $\langle P_{LZ} \rangle \approx 2\pi \left(\Delta_{SO}^2 + \sigma_{HF}^2\right)/\hbar\beta$. Such a result can be understood because the effect of detuning noise is reduced on short time scales. Supplementary Fig. 1 demonstrates this idea because the analytic curves deviate significantly from the data for $\langle P_{LZ} \rangle \gtrsim 0.2$, but for $0 < \langle P_{LZ} \rangle < 0.1$, the analytic results agrees well with the data. Based on additional simulations, we estimate the systematic error in the deduced value of σ_{ST} as obtained by fitting measured values of $\langle P_{LZ} \rangle$ to a straight line for values of β such that $0 < \langle P_{LZ} \rangle < 0.1$ to be small for most of the experimental conditions as shown in Supplementary Fig. 2.

We note that the coupling $\Delta_{ST}(t) = \delta B_\perp(t) \cos\theta + \Omega_{SO} \sin\phi \sin\theta$ depends on ϵ through the singlet mixing angle θ. This dependence means that during a LZ sweep, the coupling $\Delta_{ST}(t)$ varies both due to the linear ϵ sweep as well as charge noise. We estimate that $\frac{dE(\epsilon)}{d\epsilon} \geq 40 \frac{d\sigma_{ST}}{d\epsilon}$ for the fields studied here. We therefore expect detuning fluctuations to be the dominant noise source. Furthermore, when $|E(\epsilon)| < \sigma_{ST}$, σ_{ST} changes by only a few percent during the sweep and is likely not a significant source of error in the measurement of $\Delta_{ST}(t)$. Additionally, we note that the simulations in Supplementary Fig. 1, which include ϵ-dependent coupling, demonstrate that the fitting procedure described above allows an accurate measurement of σ_{ST}. Finally, we have also performed additional simulations, taking into account the measured values of $E(\epsilon)$, which deviate slightly from the values predicted by assuming a constant tunnel coupling, and we observe no significant change in our results.

SUPPLEMENTARY NOTE 2. DERIVATION OF NUCLEAR POLARIZATION CHANGE $\langle \delta m \rangle$

Here we derive equations (4) and (5) in the main text. Let $\Delta_{ST} = \Delta_{SO} + \Delta_{HF}$ where Δ_{SO} is real and

$$\Delta_{HF} = \sum_j \lambda_j I_j^+, \tag{7}$$

where I_j^+ is the raising operator for the jth nuclear spin, and the λ_j are individual coupling constants. We assume that there are many nuclear spins, so that each coupling constant is small. Also,

$$\sigma_{HF}^2 \equiv \langle |\Delta_{HF}^2| \rangle = \frac{2}{3} I(I+1) \sum_j \lambda_j^2 = \frac{5}{2} \sum_j \lambda_j^2, \tag{8}$$

where $I = \frac{3}{2}$ is the spin of the nuclei, and the angular brackets refer to an average over the distribution of nuclear spins.

We pick one of the nuclear spins, j, and we wish to compute $\langle \delta m_j \rangle$, the mean value of the change in I_j^z after one sweep. Let $P_{LZ}(\Delta_{ST})$ be the probability of an $S - T_+$ transition for a fixed value of Δ_{HF}. Clearly, P_{LZ} depends on $|\Delta_{ST}|$. We calculate δm_j as follows. Write

$$\Delta_{ST} = a + be^{i\theta_j}, \tag{9}$$

where a includes the contributions of spin orbit and of all nuclei other than the nucleus j, and the second term represents the contribution (of order λ_j) from nucleus j. According to equation (31) of Ref. [4], the value of δm_j for this configuration should be given by

$$\delta m_j = \frac{1}{2\pi} \oint d\theta_j P_{LZ}(\Delta_{ST}) \frac{d\varphi}{d\theta_j}, \tag{10}$$

where $\varphi = \arctan(\mathrm{Im}(\Delta_{ST})/\mathrm{Re}(\Delta_{ST}))$ specifies the orientation of Δ_{ST} in the complex plane. Without loss of generality, we may suppose that a is real. Then we have, ignoring terms that are higher order in b/a,

$$\frac{d\varphi}{d\theta_j} = \frac{b}{a} \cos\theta_j \tag{11}$$

$$P_{LZ}(\Delta_{ST}) = P_{LZ}(a) + bP'_{LZ}(a)\cos\theta_j \tag{12}$$

$$\delta m_j = \frac{b^2}{2a} P'_{LZ}(a), \tag{13}$$

where $P'_{LZ}(a)$ is the derivative of $P_{LZ}(a)$ with respect to a. Averaging over nuclear configurations, we obtain

$$\langle \delta m_j \rangle = \langle b^2 \rangle \left\langle \frac{P'_{LZ}(a)}{2a} \right\rangle, \tag{14}$$

with $\langle b^2 \rangle = (5/2)\lambda_j^2$. In the case of no charge noise, we have

$$P_{LZ}(\Delta_{ST}) = 1 - \exp\left(-\frac{2\pi|\Delta_{ST}|^2}{\hbar\beta}\right), \tag{15}$$

so

$$\frac{P'_{LZ}(a)}{2a} = \frac{2\pi}{\hbar\beta}(1 - P_{LZ}(a)) \tag{16}$$

and

$$\langle \delta m_j \rangle = \frac{2\pi}{\hbar\beta} \langle b^2 \rangle \langle 1 - P_{LZ}(a) \rangle. \tag{17}$$

Finally, we sum over all nuclear spins and make the replacement $a \approx |\Delta_{ST}|$, obtaining

$$\langle \delta m \rangle = \frac{2\pi}{\hbar\beta}\sigma_{HF}^2 \langle 1 - P_{LZ}(\Delta_{ST})\rangle. \tag{18}$$

The collapse demonstrated in Fig. 4(c) in the main text can be understood from Supplementary Equation (18), assuming constant Δ_{ST} and fixed probability. In this case, $\beta \propto |\Delta_{ST}|^2$ from Supplementary Equation (15), and hence $\langle \delta m \rangle \propto \sigma_{HF}^2/|\Delta_{ST}|^2$.

In the case of a fixed splitting, Supplementary Equation (18) reduces to

$$\langle \delta m \rangle = \frac{2\pi}{\hbar\beta}\sigma_{HF}^2 \exp\left(-\frac{2\pi|\Delta_{ST}|^2}{\hbar\beta}\right). \tag{19}$$

In Supplementary Equation (19), $\langle \delta m \rangle \to 0$ for both $\beta \to 0$ and $\beta \to \infty$. In practice however, experiments necessarily average over the hyperfine distribution. Thus, using Supplementary Equation (4) with $\Delta_{SO} = 0$ to compute $\langle 1 - P_{LZ}(\Delta_{ST})\rangle$, we have

$$\langle \delta m \rangle = \frac{2\pi}{\hbar\beta}\sigma_{HF}^2 Q \tag{20}$$

$$= \frac{\frac{2\pi\sigma_{HF}^2}{\hbar\beta}}{1 + \frac{2\pi\sigma_{HF}^2}{\hbar\beta}}. \tag{21}$$

According to Supplementary Equation (21), in the limit of slow sleeps, where $\beta \to 0$, $\langle \delta m \rangle \to 1$, and in the limit of fast sweeps, where $\beta \to \infty$, $\langle \delta m \rangle \to 0$, as expected.

The theory curves in Figs. 4(c) and (d) in the main text were generated by computing Supplementary Equation (18). For each field angle ϕ, the parameters θ, Δ_{SO}, and σ_{HF} were calculated using the fitted values of the spin-orbit and hyperfine couplings as well as the measured tunnel coupling. Supplementary Equation (4) was then solved using the calculated parameters to find the rate β such that $\langle P_{LZ}\rangle = 0.4$. In order to compare with data on the dDNP rate, the theoretical curves for $\langle \delta m \rangle$ were multiplied by fitting constants C, which are different for the two curves. As explained in the main text, we expect the ratio between the dDNP rate and $\langle \delta m \rangle$ to depend on the magnetic field but to be independent of the sweep rate.

Data taken at fixed sweep rate β also show a suppression of DNP, as shown in Supplementary Fig. 4(a). In this case, $\langle P_{LZ}\rangle$ increases with $|\phi|$ because of spin-orbit coupling [Supplementary Fig. 4(b)], and $\langle P_{LZ}\rangle$ therefore increases, causing $\langle \delta m \rangle$ to decrease, according to Supplementary Equation (18).

To address the effect of charge noise on dDNP, we recompute Supplementary Equation (18) in the limit of strong noise using the results of Ref. [7], making the replacement

$P(a) = \frac{1}{2}\left(1 - \exp\left(-\frac{4\pi a^2}{\hbar\beta}\right)\right)$ for $P_{LZ}(a)$ both in the derivation leading to Supplementary Equation (18) and in Supplementary Equation (4) for the computation of β. The expected dDNP in the presence of strong noise is shown in Supplementary Fig. 5, and it does not significantly deviate from the case without noise, at least at the level of the experimental accuracy.

SUPPLEMENTARY NOTE 3. EXPECTED DNP RATE

In this section we give a simple calculation to explain the value of the peak ($\phi = 0°$) dDNP rate, as shown in Fig. 4 of the main text. Additional measurements were carried out to measure the pumping rate of the sum hyperfine field, $(B_r + B_l)/2$, where B_r and B_l denote the longitudinal hyperfine fields in the right and left dots. This rate was determined by measuring the location of ϵ_{ST} before and after a series of LZ sweeps to polarize the nuclei at $B = 0.2$ T. We observe that the sum field is pumped roughly twice as efficiently as the difference field, $\delta B_z = B_r - B_l$. Setting $(\dot{B}_r + \dot{B}_l)/2 = 2(\dot{B}_r - \dot{B}_l)$, where $\dot{B}_{l(r)}$ indicates the pumping rate of the left(right) dot, we have $\dot{B}_l = (3/5)\dot{B}_r$, meaning that the left dot is pumped 3/5 as often as the right dot. Under these conditions, the average gradient builds up at a rate (per electron spin flip) of $(\dot{B}_r - \dot{B}_l)/(\dot{B}_r + \dot{B}_l)$ that is only 1/4 the rate that would occur if nuclear spin flips occurred in only one dot.

To determine the expected change in δB_z, we require the approximate number of spins overlapped by the electronic wave function in the double dot. We have measured the inhomogeneous dephasing time of electronic oscillations around δB_z and find $T_2^* = 18$ ns [8]. This dephasing time corresponds to a rms value of the gradient $\sigma_{\delta B_z} \equiv \sqrt{\langle|\delta B_z|^2\rangle} = h/\left(|g^*|\mu_B\sqrt{2}\pi T_2^*\right) = 2$ mT, where h is Planck's constant. The total number of spins overlapped by the wavefunction is $N = \left(h_1/\sigma_{\delta B_z}\right)^2 \approx 3\times 10^6$, where $h_1 = 4.0$ T [9]. If all nuclear spins were fully polarized, then the dots would experience a hyperfine field of $h_0 = 5.3$ T [9], and if the nuclear spins in the two dots were fully polarized in opposite directions, the gradient would be $2h_0$. Therefore, the expected change in the gradient per electron spin flip, corresponding to a change in nuclear angular momentum of $\hbar$, is $\frac{2\pi}{\hbar} \times \frac{2|g^*|\mu_B h_0}{2I(N/2)} = 12$ kHz, where $I = 3/2$ is the nuclear spin. The average dDNP under actual conditions is 1/4 of this value, or 3 kHz, in reasonable agreement with our observations. In addition, we note the reasonable agreement between the measured value of $\sigma_{\delta B_z} = 2$ mT and the root-mean-square

hyperfine gap $\sqrt{\langle|\delta B_{\perp}(t)|^2\rangle} \approx 34$ neV/ $(|g^*|\mu_B) = 1.5$ mT.

SUPPLEMENTARY REFERENCES

[1] J. R. Petta, H. Lu, and A. C. Gossard, Science **327**, 669 (2010).

[2] M. D. Shulman, S. P. Harvey, J. M. Nichol, S. D. Bartlett, A. C. Doherty, V. Umansky, and A. Yacoby, Nature Communications **5**, 5156 (2014).

[3] S. Shevchenko, S. Ashhab, and F. Nori, Physics Reports **492**, 1 (2010).

[4] I. Neder, M. S. Rudner, and B. I. Halperin, Physical Review B **89**, 085403 (2014).

[5] C. Dickel, S. Foletti, V. Umansky, and H. Bluhm, (2014), arXiv:1412.4551.

[6] O. E. Dial, M. D. Shulman, S. P. Harvey, H. Bluhm, V. Umansky, and A. Yacoby, Physical Review Letters **110**, 146804 (2013).

[7] Y. Kayanuma, Journal of the Physical Society of Japan **53**, 108 (1984).

[8] J. R. Petta, A. C. Johnson, J. M. Taylor, E. Laird, A. Yacoby, M. D. Lukin, C. M. Marcus, M. P. Hanson, and A. C. Gossard, Science **309**, 2180 (2005).

[9] J. M. Taylor, J. R. Petta, A. C. Johnson, A. Yacoby, C. M. Marcus, and M. D. Lukin, Physical Review B **76**, 035315 (2007).
